# Potential and expression of carbohydrate utilization by marine fungi in the global ocean

**DOI:** 10.1186/s40168-021-01063-4

**Published:** 2021-05-11

**Authors:** Federico Baltar, Zihao Zhao, Gerhard J. Herndl

**Affiliations:** 1 Department of Functional and Evolutionary Ecology, University of Vienna, Althanstraße 14, 1090 Vienna, Austria; 2 NIOZ, Department of Marine Microbiology and Biogeochemistry, Royal Netherlands Institute for Sea Research, Utrecht University, Den Burg, AB The Netherlands; 3 Vienna Metabolomics Center, University of Vienna, Althanstraße 14, A-1090 Vienna, Austria

**Keywords:** Pelagic fungi, CAZYmes, Metagenomic, Metatranscriptomic, Global ocean, Carbon cycle

## Abstract

**Background:**

Most of the research on the cycling of carbon in the open-ocean has focused on heterotrophic prokaryotes and eukaryotic phytoplankton, but the role of pelagic fungi remains largely enigmatic.

**Methods:**

Here, we performed a global-ocean multi-omics analysis of all pelagic fungal carbohydrate-active enzymes (CAZymes), key enzymes in the carbon cycling. We studied the occurrence, expression, diversity, functional classification, and taxonomic affiliation of the genes encoding all pelagic fungal CAZymes from the epi- and mesopelagic realm.

**Results:**

Pelagic fungi are active in carbohydrate degradation as indicated by a high ratio of CAZymes transcripts per gene. Dothideomycetes in epipelagic and the Leotiomycetes in mesopelagic waters (both from the phylum Ascomycota) are the main pelagic fungi responsible for carbohydrate degradation in the ocean. The abundance, expression, and diversity of fungal CAZymes were higher in the mesopelagic than in the epipelagic waters, in contrast to the distribution pattern of prokaryotic CAZymes.

**Conclusions:**

Our results reveal a widespread utilization of different types of CAZymes by pelagic fungi, uncovering an active and hitherto largely unexplored participation of fungi in the pelagic C cycling, where pelagic prokaryotes and fungi occupy different ecological niches, and fungi becoming relatively more important with depth.

Video abstract

**Supplementary Information:**

The online version contains supplementary material available at 10.1186/s40168-021-01063-4.

## Introduction

In terrestrial and freshwater ecosystems, fungi are one of the key organism groups responsible for the cycling of plant detritus, participating in the key elemental cycles by releasing CO_2_ to the atmosphere and inorganic N and P into the soil ([17]. In the marine environment, however, fungi are associated to debris such as driftwood, other organisms or seafloor sediments [6, 13, 15, 17, 22, 29–31, 37] and only recognized to play a key role in the element cycle in deep-sea sediments [26, 27]. Yet, recent evidence suggests that fungi are present in the oceanic water column, most likely mainly associated to particles where they might exhibit a higher biomass than prokaryotes [7]. A recent global ocean analysis based on metagenomes revealed that pelagic fungi have the genomic potential to significantly contribute to marine biogeochemical cycles (primarily linked to carbohydrate, amino acid, and lipid metabolism) [23], which together with a recent report revealing fungal glycoside hydrolases transcripts in the open ocean [12] indicate that pelagic fungi seem to be particularly active in carbohydrate utilization. However, evidence of active carbohydrate utilization by pelagic fungi is restricted only to glucoside hydrolases (GH)—one family of carbohydrate active enzymes (CAZymes) [12]. Therefore, a complete integrative analysis of metagenomic with metatranscriptomic data, covering all families of enzymes involved in carbohydrate degradation, is needed in order to clearly discern the potential and actual role of pelagic fungi involved in the carbon cycling.

To fill that critical gap in knowledge, we performed a global-ocean multi-omics analysis on the presence (metagenomes-metaG) and expression (metatranscriptomes-metaT) of carbohydrate active enzymes (CAZymes). We focused on CAZymes because (i) they are the main group of enzymes involved in cleaving carbohydrates which together with proteins are the major macromolecules in organisms and in marine snow [3], (ii) we recently found CAZymes to be key enzymes in the degradation of organic matter by pelagic heterotrophic prokaryotes in the ocean [2, 40], and (iii) a large number of published microbial CAZyme gene profiles/catalogs are available from various environments [2, 14, 21, 28, 40].

## Results and discussion

We examined the occurrence, diversity, functional classification, taxonomic affiliation, and metabolic expression of genes encoding CAZymes from 445 metagenomes and 440 metatranscriptomes from 68 Tara stations of size fractions ranging from 0.8-2,000 μm, covering the epipelagic (0-200 m), and mesopelagic (200-1000 m) waters (see “Methods” section). Of the 116 million non-redundant sequences from global ocean eukaryotic genes, 80,892 eukaryotic CAZyme sequences were retrieved. Fungi-affiliated sequences contributed 3.9% (3184 out of 80,892) to these eukaryotic CAZyme sequences. The abundance of fungal CAZyme sequences are ranging from 1 to 2044 in the metagenomes and from 1 to 2623 in the metatranscriptomes, respectively. The occurrence and expression values of fungal CAZyme sequences in the metagenomic and metatranscriptomic dataset were downloaded from http://www.genoscope.cns.fr/tara/.

**Fig. 1:**
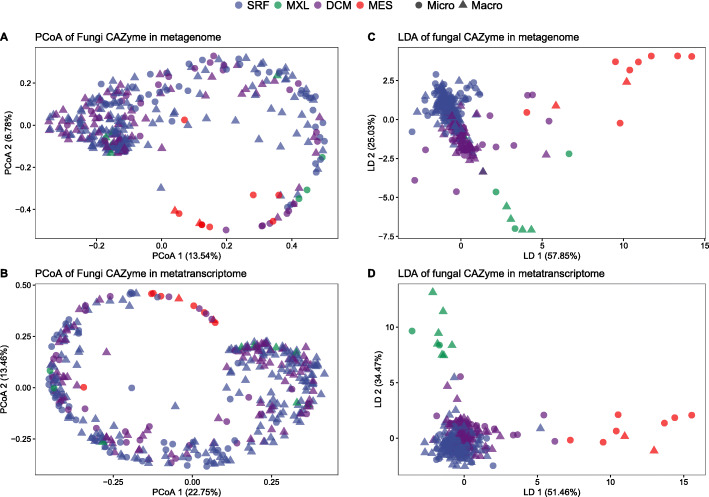
Distribution of samples and niche differentiation of genes encoding fungal CAZymes. Principal coordinate analysis (PCoA) of fungal CAZyme genes present in the metagenome (**a**) and metatranscriptome (**b**); and linear discriminate analysis (LDA) of fungal CAZyme genes present in the metagenome (**c**); and in the metatranscriptome (**d**). Micro, micro-mycobiome (0.8-5 μm); Macro, macro-mycobiome (5-2000 μm)

A principal coordinate (PCoA) and linear discriminate (LDA) analyses against Bray-Curtis dissimilarity based on the gene occurrence (metagenome) and expression (metatranscriptome) of fungal CAZyme families revealed depth-related differences in the composition of enzyme families, mostly between the epipelagic (SRF, surface layer; MXL, mixed layer; DCM, deep chlorophyll maximum layer) and mesopelagic (MES) waters for both size classes (micro- [0.8-5 μm] and macro-mycobiome [5-2,000 μm]) (Fig. 1, Ext. Fig. S2). The linear discriminate analysis [38], which accounts for the horseshoe effect [24], revealed a clearer niche separation at both, the metagenomic and metatranscriptomic level (Fig. 1c, d). This depth-related stratification found on both the gene and transcript level suggests a tight link between the genomic potential and expression of pelagic fungal CAZymes (Ext. Fig. S2). This depth-stratification of CAZymes has also been found in prokaryotes of the epi- and mesopelagic realm [40], indicating that fungi and prokaryotes share the same depth-related changes in CAZymes, possibly related to changes in the bioavailability of the organic matter with depth in the water column.

**Fig. 2:**
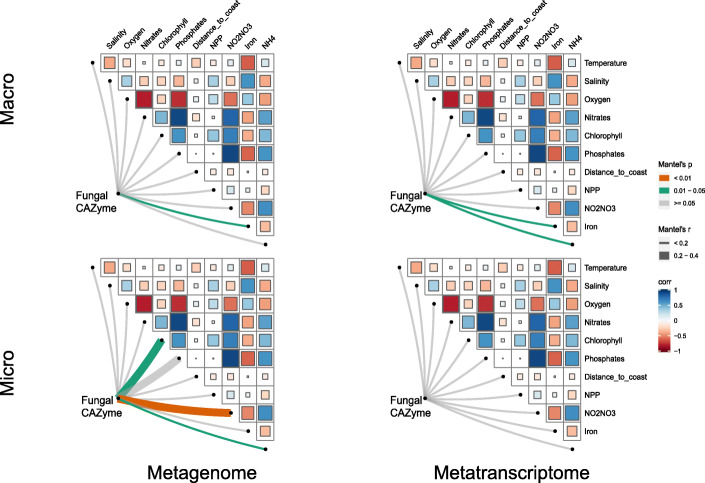
Mantel test between fungal CAZyme profile and environmental parameters. Mantel’s *r* and *p* values are indicated based on the color and the width of the connecting lines as specified in the figure legend. Micro, micro-mycobiome (0.8-5 μm); Macro, macro-mycobiome (5-2000 μm). SRF, surface; MXL, mixed layer; DCM, deep chlorophyll maximum; MES, mesopelagic

Specific environmental parameters shaped the biogeography of fungal CAZyme genes and transcripts (Fig. 2). The CAZyme gene composition of the micro-mycobiome was related to chlorophyll and nitrate+nitrate (NO_2_^−^+NO_3_^−^) and NH4^+^ concentration (which were also strongly correlated with chlorophyll), while that of the macro-mycobiome was linked to iron. In contrast, the expression of CAZymes of the micro-mycobiome was not linked to environmental parameters, but that of the macro-mycobiome was related to ammonia and iron. This suggests a link between the macro-mycobiome’s carbohydrate degradation and nitrogen (mostly ammonium) and iron availability, indicating a potential bottom-up control of the pelagic mycobiome which can be in most regions nutrient limited (mostly by N). These results contrast to the distribution pattern of CAZyme genes of pelagic prokaryotes which were linked to temperature, salinity, and oxygen [40], indicating different environmental factors control carbohydrate cleavage of prokaryotes and fungi in the ocean.

**Fig. 3:**
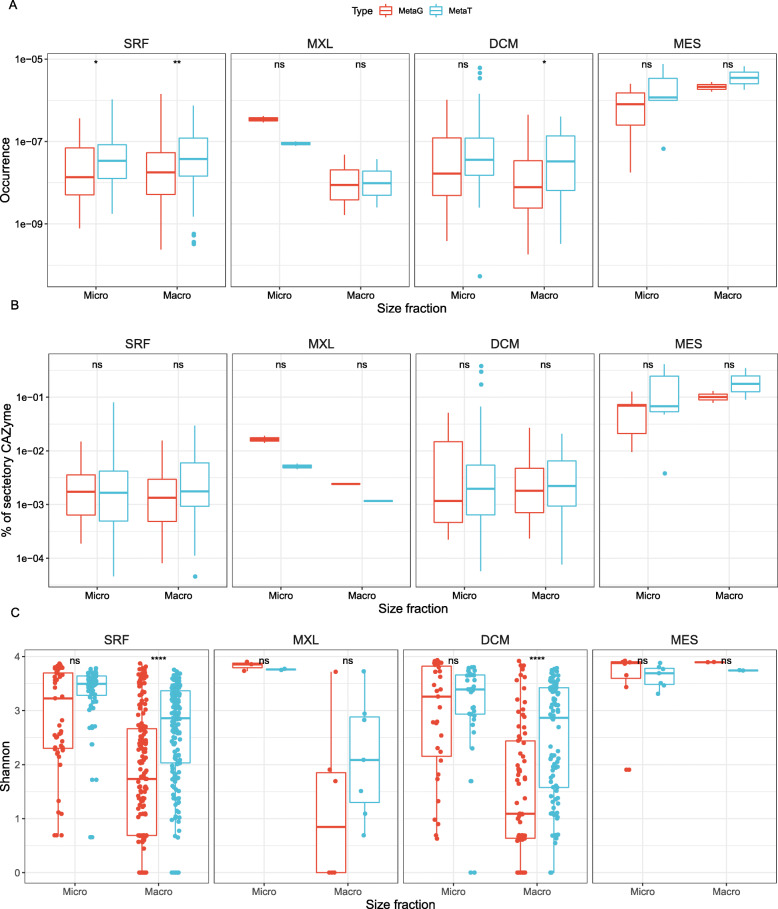
Occurrence (**a**), secretory capacity (**b**), and α-diversity (**c**) of genes and transcripts for fungal CAZymes. Micro, micro-mycobiome (0.8-5 μm); Macro, macro-mycobiome (5-2000 μm). Box shows median and interquartile range (IQR); whiskers show 1.5 × IQR of the lower and upper quartiles or range; outliers extend to the data range. Statistics are based on Wilcoxon test, *P<0.05, **P<0.01, ***P<0.001, ****P<0.0001; ns, not significant. SRF, surface; MXL, mixed layer; DCM, deep chlorophyll maximum; MES, mesopelagic

The abundance of fungal CAZyme transcripts was equal or even higher (except for the micro-mycobiome of the mixed layer [MXL]) than that of genomic fungal CAZyme sequences at all depths and in all size fractions, indicating that the fungal community is highly active in cleaving carbohydrates (Fig. 3a). Yet, caution is needed when interpreting patterns for MXL since the available number of samples for that depth range in the database was limited. The abundances of fungal CAZyme genes and transcripts were higher in the mesopelagic than in the epipelagic (Fig. 3a). This is in agreement with recent evidence suggesting that fungal biomass [7] and the relative contribution of fungal to total microbial genomic sequences are relatively higher in mesopelagic than in epipelagic waters [23]. Collectively, this indicates that with increasing depth, fungi play an increasingly important role in the degradation of organic matter.

CAZymes cannot only be located in the cytoplasmic space but also secreted into the periplasmic space (cell-associated CAZyme) or into the ambient environment (cell-free CAZyme). We also investigated the prevalence and origin of secreted CAZymes in the pelagic mycobiome (Fig. 3b), since the capability of secreting cell-free CAZymes has been recently recognized as a widespread and important feature for oceanic prokaryotes linked to a preferential particle-attached lifestyle [40]. The proportion of the secretory to the total (i.e., cytosol plus secreted) fungal CAZyme pool was higher in the meso- than in the epipelagic waters (Fig. 3b). This depth-related increase in the relative proportion of secretory CAZymes was also reported for prokaryotes [40], and it is consistent with genomic evidence and extracellular enzymatic activities determined by substrate analogs [5]. This finding does not only confirm but also expands the predominance of secretory enzymatic activities in the dark ocean [4] by also incorporating fungi as another organism group contributing to the pool of dissolved (i.e., cell-free) extracellular CAZymes in the ocean.

The Shannon diversity of fungal CAZymes was generally higher (for both genes and transcripts) for the micro-mycobiome than for the macro-mycobiome in the epipelagic (SRF, MXL, and DCM), but not in the mesopelagic realm (Fig. 3c). As observed with the gene occurrence of total fungal CAZymes (Fig. 3a) and the proportion of genes encoding secretory CAZymes (Fig. 3b), the diversity of fungal CAZyme transcripts (metaT) was equal or higher to that of the genes (metaG) (Fig. 3c). The diversity of the genes and transcripts (of both size fractions) for CAZymes was generally higher in the mesopelagic than in the epipelagic, which is in contrast to the decrease in CAZyme diversity of prokaryotic origin with depth [40]. This relative increase in the diversity of fungal relative to prokaryotic CAZymes with depth might be another indication of a niche differentiation or specialization between pelagic fungi and prokaryotes. This differentiation between pelagic fungi and prokaryotes with depth might be related to a preferential association of fungi to particle where fungi might have a competitive advantage over prokaryotes, which due to their smaller size (and higher surface/volume ratio) would do relatively better than fungi in the free-living pool.

**Fig. 4:**
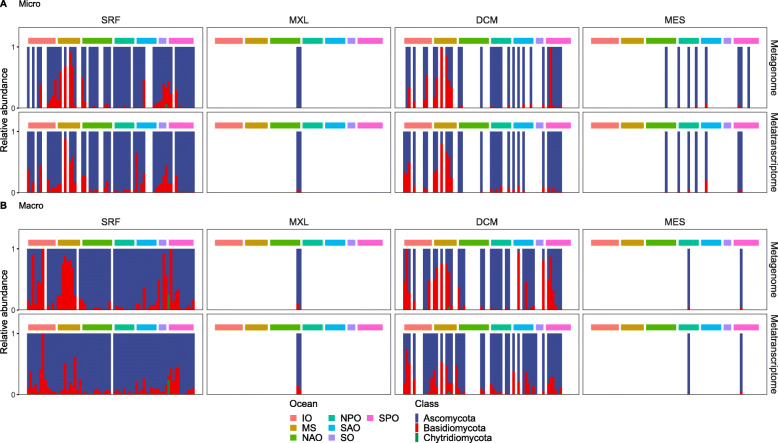
Taxonomic affiliation of genes (**a**) and transcripts (**b**) encoding fungal CAZymes at the phylum level. Micro, micro-mycobiome (0.8-5 μm); Macro, macro-mycobiome (5-2000 μm). Each bar represents a sample collected in each of the stations/location and depth; so that missing bars (empty white space) represents stations/locations where samples were not collected at that particular depth. SRF, surface; MXL, mixed layer; DCM, deep chlorophyll maximum; MES, mesopelagic; IO, Indian Ocean; MS, Mediterranean Sea; NAO, North Atlantic Ocean; North Pacific Ocean; SAO, South Atlantic Ocean; SO, Southern Ocean; SPO, South Pacific Ocean

The taxonomic affiliation of the total pelagic fungal CAZyme genes and transcripts was dominated in both size classes and all depths and locations by two phyla: the Ascomycota and the Basidiomycota (Fig. 4). This is consistent with the only previous investigation on pelagic fungal CAZymes, although that study was restricted only to glucoside hydrolases (GH)—one single family of carbohydrate active enzymes (CAZymes) [12]. This is consistent with a global ocean metagenomic study which showed that these two fungal phyla comprised >97% of the fungal reads in the epi- and mesopelagic [23]. Our study expands these findings from the genomic potential (metagenome) to the expression (metatranscriptome) level. CAZyme genes and transcripts from the phylum Chytridiomycota were also detected, but only in low proportions and restricted to specific locations, i.e., the Southern Ocean and the epipelagic layer. This is in agreement with the only available time series study (based on the 18S rRNA) of coastal fungal communities, which showed that Ascomycota and Basidiomycota dominated most of the time with only sporadic contributions of Chytridiomycota associated to phytoplankton blooms [33]. Also, Chytridiomycota CAZyme genes and transcripts were found to be relatively more abundant in cold environments such as the Southern Ocean than in tropical waters, in agreement with high Chytridiomycota abundances in sea ice and Artic waters [19, 20]. Interestingly, Chytridiomycota were not detected in the mesopelagic, which is consistent with their presumed role as parasites of diatoms [18]. Also, the presence of Basidiomycota-affiliated CAZyme genes and transcripts decreased from the epipelagic to the mesopelagic, indicating an almost exclusive role of Ascomycota in the carbohydrate degradation in the dark ocean.

At the class level, the dominant taxa affiliated to fungal CAZymes changed with size fraction, depth, and location (Ext. Fig. S3). In the epipelagic waters, the main classes of Ascomycota were Dothideomycetes and Sordariomycetes, whereas the dominant Basidiomycota CAZymes were of Malasseziomycetes origin (Ext. Fig. S3). The micro-mycome CAZymes were relatively enriched in Sordariomycetes and Eurotiomycetes compared to the macro-mycobiome, which was more enriched in Dothideomycetes (Ext. Fig. S3). Ascomycota strongly dominated in the large oceanic basins (N and S Atlantic and Pacific), whereas Basidiomycota were relatively more abundant in the Mediterranean Sea, and the Indian and Southern Ocean. The dominant Ascomycota class in the epipelagic, the Dothideomycetes, was replaced by the Leotiomycetes in the mesopelagic (Ext. Fig. S3), indicating a taxonomic shift (and ecological partitioning) between these two Ascomycota classes with depth. Whether the relative increase in Ascomycota with depth is real or due to sampling artifacts (i.e., less fungal biomass collected in deep waters could cause the most abundant member to be more detected and vice versa) would benefit from further investigation. The changes observed in the genomic potential were also found at the transcript level. Yet, the overall relation between the genomic potential and expression patterns indicates that the fungi in the different regions and depths are participating in the degradation of carbohydrates.

The secretory fraction of CAZymes were produced by the same fungal taxa as the total CAZymes, with the exception that Chytridiomycota are apparently not producing secretory CAZymes (Ext. Fig. S4 and S5). This suggests that pelagic oceanic Chytridiomycota might use a different ecological strategy compared to the dominating pelagic fungi Ascomycota and Basidiomycota by not releasing CAZymes into the environment. Parasitic and sapotrophic plant fungi have been reported to exhibit different CAZymes [34, 41], suggesting a link between ecological and nutritional strategies. Our analyses show that the genomic potential and expression of secreted CAZymes are a common feature of the CAZyme-harboring pelagic fungi (except the parasitic Chytridiomycota), consistent with the CAZyme pattern found in pelagic prokaryotes [40]. Extending the foraging theory described for oceanic prokaryotes [36] to oceanic fungi, the high contribution of hydrolytic secretory CAZymes among fungi would imply a preferential particle-attached lifestyle of pelagic fungi. This conclusion is consistent with recent findings of high fungal biomass in deep-water marine snow [7].

**Fig. 5:**
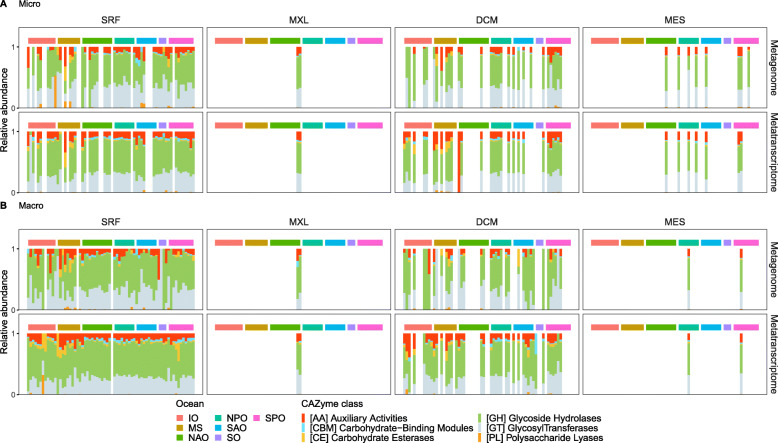
Functional classification of genes (**a**) and transcripts (**b**) encoding fungal CAZymes. Micro, micro-mycobiome (0.8-5 μm); Macro, macro-mycobiome (5-2000 μm). Each bar represents a sample collected in each of the stations/location and depth, so that missing bars (empty white space) represents stations/locations where samples were not collected at that particular depth. SRF, surface; MXL, mixed layer; DCM, deep chlorophyll maximum; MES, mesopelagic; IO, Indian Ocean; MS, Mediterranean Sea; NAO, North Atlantic Ocean; North Pacific Ocean; SAO, South Atlantic Ocean; SO, Southern Ocean; SPO, South Pacific Ocean

To decipher the specific functional roles of pelagic fungal CAZymes and how they are distributed among the different size fractions, locations, and depths, we functionally classified the fungal CAZymes (Fig. 5). The types of CAZyme functions were remarkably similar among the size fractions, locations, and depths, similar to the patterns observed for pelagic prokaryotes [40]. The dominant fungal CAZymes were glucoside hydrolases (GH) and glycosyl transferases (GT) followed by auxiliary activities (AA). While ca. 1/3 of the relative abundance of prokaryotic CAZyme genes are made up of carbohydrate esterases (CE) and carbohydrate-binding modules (CBM) [40], these CAZymes (CE and CBM) were not abundant in pelagic fungi. A significant contribution of fungal CE was detected only in the Mediterranean Sea. Further research is needed to understand the reasons for the peculiarities observed in the Mediterranean Sea, which might be associated to the characteristic P-limited conditions and/or the relatively higher temperatures observed in the deep Mediterranean waters. These results suggest different ecological and biogeochemical roles of pelagic fungi and prokaryotes in the processing of carbohydrates, where CE and CBM are almost exclusively used by prokaryotes while fungi rely mostly on GH, GT, and AA.

The functional classification of the secretory fungal CAZyme genes and transcripts was very similar to that of the total fungal CAZymes, except for the complete lack of CE (Ext. Fig. S6). This indicates that oceanic pelagic fungi do not secrete CE, and apparently are generally not relying on CE. While GT comprised around 40% of all the total prokaryotic CAZymes, secreted GT were barely detected in pelagic prokaryotes [40]. In contrast, GT were present in both the total and the secreted CAZyme pool in pelagic fungi, consistent with a previous study on marine sediment fungi [28]. As cytoplasmic GTs are involved in sugar bond formation, secretory GTs can be expected as fungi use chitin to build up the cell wall [8] requiring integral membrane chitin synthase (GT2). This is confirmed by the frequent detection of GT2 type sequences in our data (Table S1). Indeed, the presence and expression of genes encoding periplasmic GTs indicate that fungi are metabolically active in the oceanic water column. These results also imply that pelagic prokaryotes and fungi follow different strategies in using carbohydrates as indicated by the differences in their secreted CAZyme pools.

To obtain insights into the functional diversity of pelagic fungal CAZymes, we also looked into the occurrence of genes and transcripts of fungal CAZyme targeting different types of carbohydrates originating from animal, plant cell wall, and fungal and bacterial (peptidoglycan) detritus (see “Methods” section). We found similar patterns for the total (Ext. Fig. S7) and secretory (Ext. Fig. S8) fungal CAZymes at both the metagenomic and metatranscriptomic levels. The mesopelagic layers exhibited the highest abundance and expression for all groups of fungal CAZymes (i.e., targeting carbohydrates of animal, fungal and plant cell wall origin). Peptidoglycan degrading genes and transcripts (i.e., GH23, peptidoglycan lyase; GH24/GH25, lysozyme; GH73, peptidoglycan hydrolase with endo-β-N-acetylglucosaminidase specificity; GH102/GH103/GH104, peptidoglycan lytic transglycosylase; GH108, N-acetylmuramidase), however, were completely lacking (Ext. Fig. S8). This might indicate that although pelagic prokaryotes can recycle fungal-produced carbohydrates [40], pelagic fungi cannot recycle cell debris from bacteria. Caution should be paid, however, since domain-similarity based analysis as done here might lead to biases if the database is limited. Hence, the lack of fungal CAZymes to recycle bacterial cell wall components requires further investigations.

## Conclusions

In concert, our results reveal that oceanic pelagic fungi are active contributors to the cycling of carbohydrates in all ocean basins and depths, and not just passively advected spores. The ability to secrete CAZymes is a relevant and widespread ecological feature in pelagic fungi, as observed also for prokaryotes, particularly in the dark ocean. We identified the main groups of pelagic fungi (Ascomycota and Basidiomycota) responsible for carbohydrate degradation in the ocean and revealed important biogeographical (e.g., peculiarities of the Mediterranean Sea) and depth-related (e.g., epi- vs. mesopelagic layer) differences. The environmental parameters affecting the distribution of fungal and prokaryotic CAZymes are different, suggesting distinct environmental drivers for pelagic fungal versus prokaryotic CAZyme activities. The relative abundance of specific types of pelagic fungal CAZymes also differs from that of prokaryotes, pointing toward distinct ecological niches of these two groups. There is evidence that the relative importance of fungi in carbohydrate processing increases with depth. Further research on the carbon cycling of pelagic fungi, specifically comparing free-living to particle-associated communities will provide a more complete picture of the role of pelagic fungi in the ocean. Overall, from our study, we can conclude that pelagic fungi play most likely a major role in the cycling of carbohydrates in the global ocean.

## Materials and methods

The sequences and occurrences in the corresponding metagenomes and metatranscriptomes of marine eukaryotic genes were downloaded from literature [11]. The accession number for is PRJEB4352 for the metagenomics data and PRJEB6609 for the metatranscriptomics data. Gene occurrence in metagenomic and metatranscriptomic dataset is available at http://www.genoscope.cns.fr/tara/ “Tara Oceans Eukaryote Gene Catalog.” Environmental parameters were downloaded from the original paper (Supplementary Data 5, [11]), the mantel test was perform between fungal CAZyme beta-diversity and environmental parameters to test the driving factors which shapes fungal CAZyme in marine environments. We use the presence of signal peptides as a proxy for secretory CAZymes because signal peptides are short amino acid sequences in the amino terminus of proteins that direct proteins into, or across, membranes, thus the CAZymes enconding genes containing signal peptide sequences can be probably translocated from cytoplasmic to the periplasmic space or outside the cell. To identify CAZyme-like sequences, the 116 million eukaryotic genes were first compared against the dbCAN database [39] [39] (dbCAN HMMdb release 8.0) using DIAMOND version.0.8.36 blast [9] (*e* value < 1 × 10^−102^). Sequences with positive hits were extracted for taxonomic identification using the lowest common ancestor algorithm adapted from DIAMOND v.0.8.36 [9] blast by searching against the NCBI non-redundant (NR, downloaded in March 2020) database. The top 10% hits with an *e* value < 1 × 10^−5^ were used for taxonomic affiliation assessment (--top 10). Only sequences annotated as Ascomycota, Basidiomycota, Mucoromycota, or Chytridiomycota at the phylum level were detected. Those sequences were further annotated with dbCAN metaSever (http://bcb.unl.edu/dbCAN2/) using three algorithms and very conservative thresholds: DIAMOND (*e* value < 1 × 10^−102^), HMMER (*e* value < 1e−^17^ and coverage > 0.45), and Hotpep (frequency > 2.6, hits > 6); sequences with positive hits from at least 2 prediction tools were admitted and results from HMMER annotation were kept as CAZyme functional classification, followed by results from DIAMOND if there was no HMMER annotation. For example, if a sequence is annotated as GH43_5 by HMMER but GH47 by Diamond blast, GH43_5 was assigned to the sequences as its potential function. The functional annotations at the CAZyme family level were further grouped into CAZyme class level according to the common designations from CAZyme database (www.cazy.org). The fungal CAZyme sequences analyzed in this study and the corresponding annotations gene occurrence can be found in supplementary files (Supplementary Dataset 1, Table S1). SignalP [1] (5.0) was used to detect the presence of signal peptides for fungal sequences under eukaryotic mode. CAZyme families targeting different carbohydrate sources were identified according to previous reports [10]. The sub-families were grouped into families and assigned to substrate targets (Table S1). Size fractions were defined as micro-mycobiome for samples from 0.8-5 μm (0.8-3 μm was used for samples from the mesopelagic waters as it was the only available range) and macro-mycobiome for samples from 5-2000 μm (3-2000 μm was used for samples from mesopelagic waters). Data analysis was performed using R project (R version 3.6.1, www.R-project.com). *Vegan* [25], *rtk* [32], and *ggplot2* [16] were used for ordination, diversity calculation, and visualization, respectively. The LDA analyses were performed by *MASS* [35]. LDA is a supervised ordination method. We use this method to compare with PCoA ordination results (which is an un-supervised method) to avoid the “horse-shoe” effect [24].

## Supplementary Information

**Additional file 1: Supplementary Dataset 1 and Table S1.** Fungal CAZyme sequences analyzed in this study (Dataset 1) and the corresponding annotations gene occurrence (Table S1). **Table S1.** Information on the CAZyme subfamilies grouping and abundance table (enclosed Excel file containing 2 sheets). Sheet 1 ("cazyme.substrate"): contains the information of the grouping of CAZymes sub-families and assigned substrate targets. Sheet 2 (" abundance_table"): contains the abundance table of CAZymes in all samples.

**Additional file 2: Figure S1.** Distribution of samples and niche differentiation of genes encoding fungal CAZymes categorized by “depth” (top plots) and by “size” (lower plots). Principal coordinate analysis (PCoA) of fungal CAZyme genes present in the metagenome (left plots) and metatranscriptome (right plots). Micro, micro-mycobiome (0.8-5 μm); Macro, macro-mycobiome (5-2,000 μm). **Figure S2.** Correlation of the occurrence of the metagenome and metatranscriptome of genes encoding fungal CAZymes for the macro-mycobiome (left) and micro-mycobiome (right). P-values and R^2^ provided in the plots. Micro, micro-mycobiome (0.8-5 μm); Macro, macro-mycobiome (5-2,000 μm). **Figure S3.** Taxonomic affiliation of genes (A) and transcripts (B) encoding fungal CAZymes at the class level. Micro, micro-mycobiome (0.8-5 μm); Macro, macro-mycobiome (5-2,000 μm). Each bar represents a sample collected in each of the stations/location and depth; so that missing bars (empty white space) represents stations/locations where samples were not collected at that particular depth. SRF, surface; MXL, mixed layer; DCM, deep chlorophyll maximum; MES, mesopelagic; IO, Indian Ocean; MS, Mediterranean Sea; NAO, North Atlantic Ocean; North Pacific Ocean; SAO, South Atlantic Ocean; SO, Southern Ocean; SPO, South Pacific Ocean. **Figure S4.** Taxonomic affiliation of genes (A) and transcripts (B) encoding secretory fungal CAZymes at the phylum level. Micro, micro-mycobiome (0.8-5 μm); Macro, macro-mycobiome (5-2,000 μm). Each bar represents a sample collected in each of the stations/location and depth; so that missing bars (empty white space) represents stations/locations where samples were not collected at that particular depth. SRF, surface; MXL, mixed layer; DCM, deep chlorophyll maximum; MES, mesopelagic; IO, Indian Ocean; MS, Mediterranean Sea; NAO, North Atlantic Ocean; North Pacific Ocean; SAO, South Atlantic Ocean; SO, Southern Ocean; SPO, South Pacific Ocean. **Figure S5.** Taxonomic affiliation of genes (A) and transcripts (B) encoding secretory fungal CAZymes at the class level. Micro, micro-mycobiome (0.8-5 μm); Macro, macro-mycobiome (5-2,000 μm). Each bar represents a sample collected in each of the stations/location and depth; so that missing bars (empty white space) represents stations/locations where samples were not collected at that particular depth. SRF, surface; MXL, mixed layer; DCM, deep chlorophyll maximum; MES, mesopelagic; IO, Indian Ocean; MS, Mediterranean Sea; NAO, North Atlantic Ocean; North Pacific Ocean; SAO, South Atlantic Ocean; SO, Southern Ocean; SPO, South Pacific Ocean. **Figure S6.** Functional classification of genes (A) and transcripts (B) encoding secretory fungal CAZymes. Micro, micro-mycobiome (0.8-5 μm); Macro, macro-mycobiome (5-2,000 μm). Each bar represents a sample collected in each of the stations/location and depth; so that missing bars (empty white space) represents stations/locations where samples were not collected at that particular depth. SRF, surface; MXL, mixed layer; DCM, deep chlorophyll maximum; MES, mesopelagic; IO, Indian Ocean; MS, Mediterranean Sea; NAO, North Atlantic Ocean; North Pacific Ocean; SAO, South Atlantic Ocean; SO, Southern Ocean; SPO, South Pacific Ocean. **Figure S7.** Occurrence of genes and transcripts for fungal CAZymes targeting different carbohydrate sources. Micro, micro-mycobiome (0.8-5 μm); Macro, macro-mycobiome (5-2,000 μm). Box shows median and interquartile range (IQR); whiskers show 1.5 × IQR of the lower and upper quartiles or range; outliers extend to the data range. Statistics are based on a Wilcoxon test, *P<0.05, **P<0.01, ***P<0.001, ****P<0.0001, ns, not significant. SRF, surface; MXL, mixed layer; DCM, deep chlorophyll maximum; MES, mesopelagic. Note that carbohydrates originating from bacterial (peptidoglycan) detritus were not plotted because they were not found in the metagenome or metatranscriptomes of pelagic fungi. **Figure S8.** Occurrence of genes and transcripts for secretory fungal CAZymes targeting different carbohydrate sources. Micro, micro-mycobiome (0.8-5 μm); Macro, macro-mycobiome (5-2000 μm). Box shows median and interquartile range (IQR); whiskers show 1.5 × IQR of the lower and upper quartiles or range; outliers extend to the data range. Statistics are based on Wilcoxon test, *P<0.05, **P<0.01, ***P<0.001, ****P<0.0001, ns, not significant. SRF, surface; MXL, mixed layer; DCM, deep chlorophyll maximum; MES, mesopelagic.

**Additional file 3.**

## Data Availability

All data used in this work is publicly available. The sequences and occurrences in the corresponding metagenomes and metatranscriptomes of marine eukaryotic genes were downloaded from Carradec et al., a global ocean atlas of eukaryotic genes. *Nature Communications* **9**, 373, doi:10.1038/s41467-017-02342-1 (2018). We also provide the sequences we analyzed in the supplementary material (as Supplementary Dataset 1) for easier direct access.
