# Supplementary material for: Potential and expression of carbohydrate utilization by marine fungi in the global ocean
Source: Microbiome. 2021 May 11;9:106. doi: 10.1186/s40168-021-01063-4 (PMC8114511; doi:10.1186/s40168-021-01063-4)
Supplement: Supplementary file 3 — Additional file 2: Figure S1. Distribution of samples and niche differentiation of genes encoding fungal CAZymes categorized by “depth” (top plots) and by “size” (lower plots). Principal coordinate analysis (PCoA) of fungal CAZyme genes present in the metagenome (left plots) and metatranscriptome (right plots). Micro, micro-mycobiome (0.8-5 μm); Macro, macro-mycobiome (5-2,000 μm). Figure S2. Correlation of the occurrence of the metagenome and metatranscriptome of genes encoding fungal CAZymes for the macro-mycobiome (left) and micro-mycobiome (right). P-values and R2 provided in the plots. Micro, micro-mycobiome (0.8-5 μm); Macro, macro-mycobiome (5-2,000 μm). Figure S3. Taxonomic affiliation of genes (A) and transcripts (B) encoding fungal CAZymes at the class level. Micro, micro-mycobiome (0.8-5 μm); Macro, macro-mycobiome (5-2,000 μm). Each bar represents a sample collected in each of the stations/location and depth; so that missing bars (empty white space) represents stations/locations where samples were not collected at that particular depth. SRF, surface; MXL, mixed layer; DCM, deep chlorophyll maximum; MES, mesopelagic; IO, Indian Ocean; MS, Mediterranean Sea; NAO, North Atlantic Ocean; North Pacific Ocean; SAO, South Atlantic Ocean; SO, Southern Ocean; SPO, South Pacific Ocean. Figure S4. Taxonomic affiliation of genes (A) and transcripts (B) encoding secretory fungal CAZymes at the phylum level. Micro, micro-mycobiome (0.8-5 μm); Macro, macro-mycobiome (5-2,000 μm). Each bar represents a sample collected in each of the stations/location and depth; so that missing bars (empty white space) represents stations/locations where samples were not collected at that particular depth. SRF, surface; MXL, mixed layer; DCM, deep chlorophyll maximum; MES, mesopelagic; IO, Indian Ocean; MS, Mediterranean Sea; NAO, North Atlantic Ocean; North Pacific Ocean; SAO, South Atlantic Ocean; SO, Southern Ocean; SPO, South Pacific Ocean. Figure S5. Taxonomic affiliation of genes ( [file 40168_2021_1063_MOESM3_ESM.docx]

**Supplementary Information for**

*Potential and expression of carbohydrate utilization by marine fungi in the global ocean.*

Federico Baltar^1*^, Zihao Zhao^1^, Gerhard J. Herndl^1,2,3^

^1^Department of Functional and Evolutionary Ecology, University of Vienna, Althanstraße 14, 1090 Vienna, Austria

^2^ NIOZ, Department of Marine Microbiology and Biogeochemistry, Royal Netherlands Institute for Sea Research, Utrecht University, AB Den Burg, The Netherlands

^3^ Vienna Metabolomics Center, University of Vienna, Althanstraße 14, A-1090 Vienna, Austria

* Correspondence: Federico Baltar

**Email:**  [federico.baltar@univie.ac.at](mailto:xxxxx@xxxx.xxx)

**This PDF file includes:**

Ext. Figures S1-S8

**Other supplementary materials for this manuscript include the following:**

Table S1

Supplementary Dataset 1


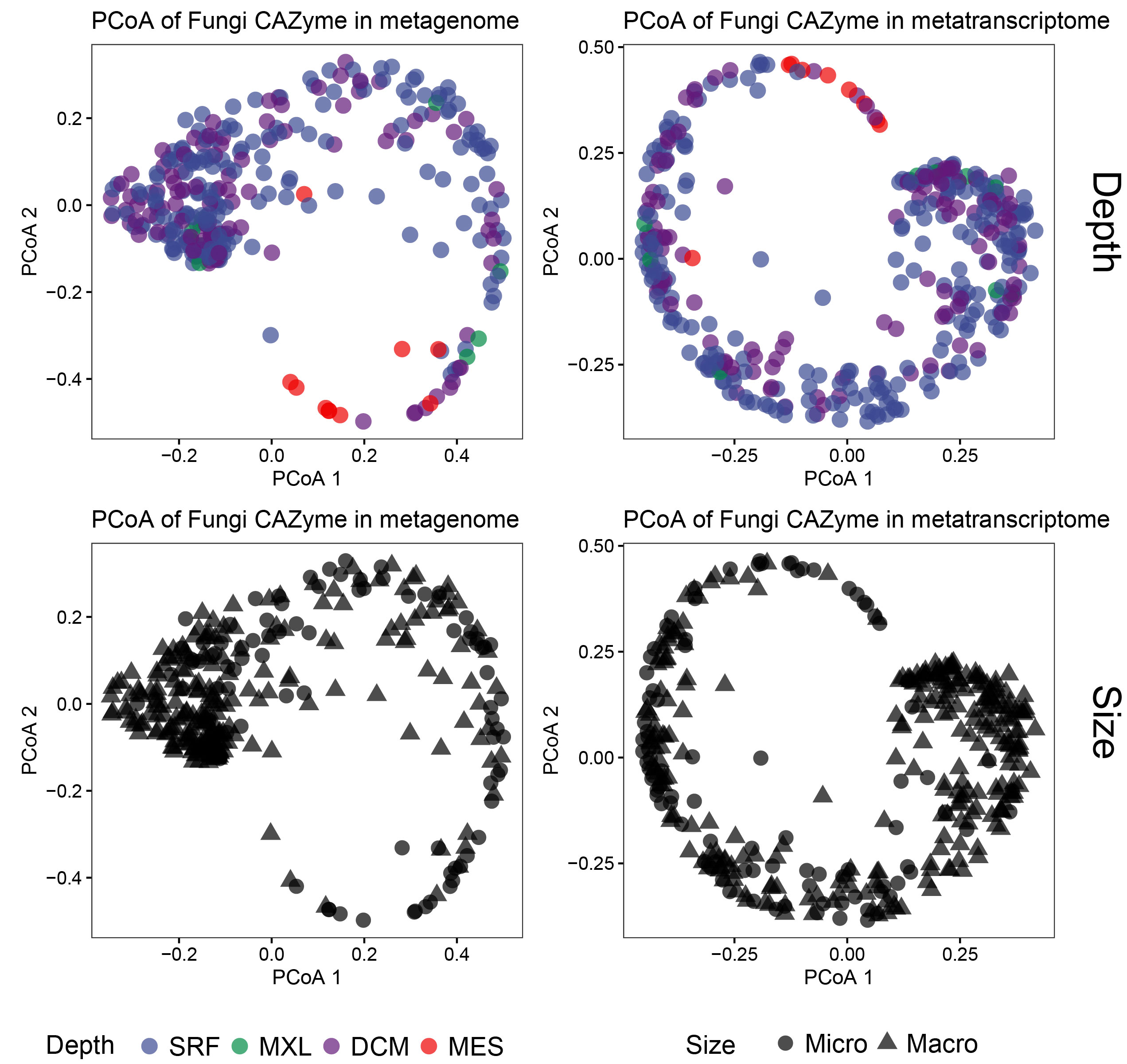


**Figure S1.** Distribution of samples and niche differentiation of genes encoding fungal CAZymes categorized by “depth” (top plots) and by “size” (lower plots). Principal coordinate analysis (PCoA) of fungal CAZyme genes present in the metagenome (left plots) and metatranscriptome (right plots). Micro, micro-mycobiome (0.8-5 µm); Macro, macro-mycobiome (5-2,000 µm).


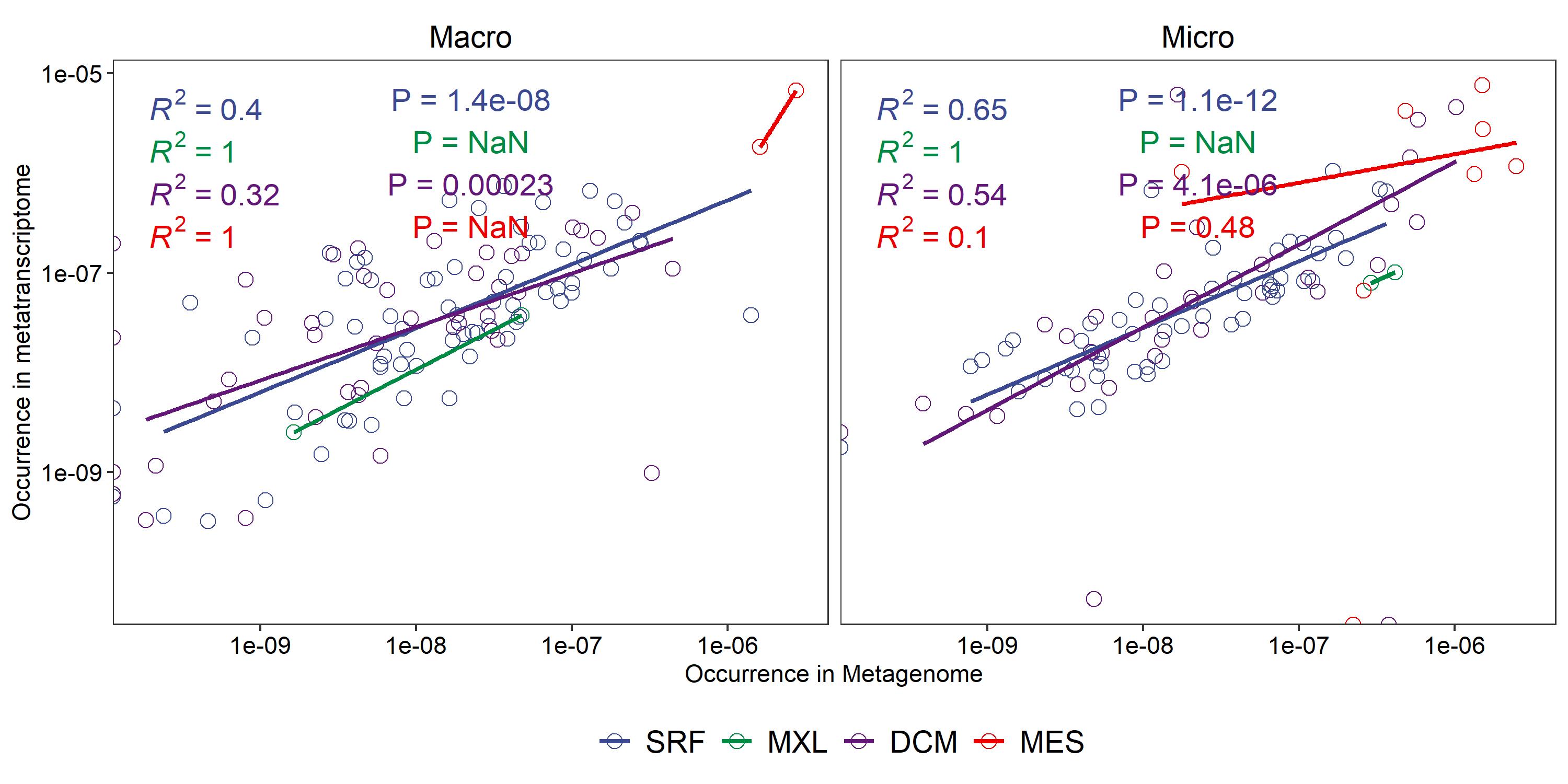


**Figure S2.** Correlation of the occurrence of the metagenome and metatranscriptome of genes encoding fungal CAZymes for the macro-mycobiome (left) and micro-mycobiome (right). P-values and R^2^ provided in the plots. Micro, micro-mycobiome (0.8-5 µm); Macro, macro-mycobiome (5-2,000 µm).


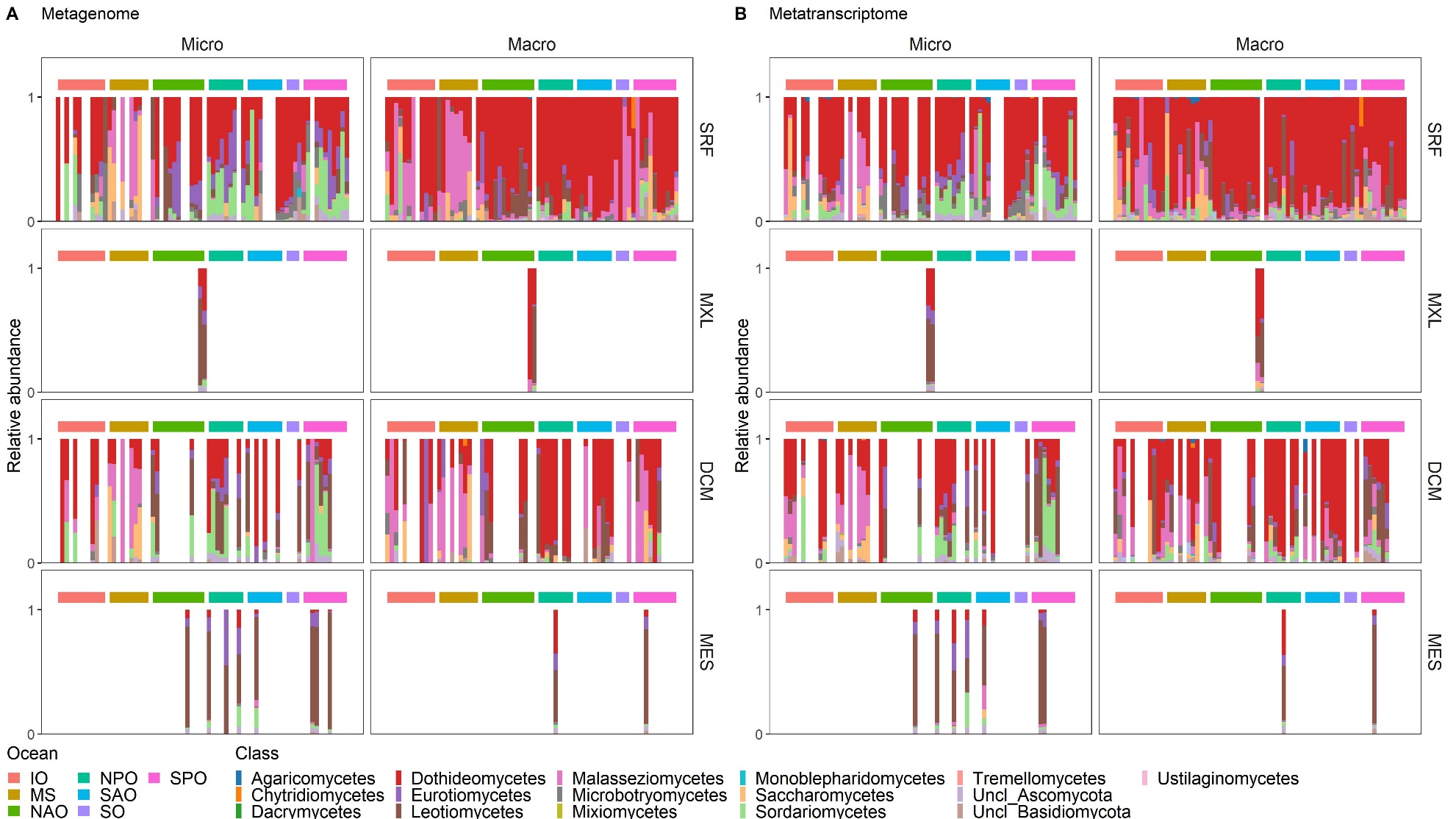


**Figure S3.** Taxonomic affiliation of genes (A) and transcripts (B) encoding fungal CAZymes at the class level. Micro, micro-mycobiome (0.8-5 µm); Macro, macro-mycobiome (5-2,000 µm). Each bar represents a sample collected in each of the stations/location and depth; so that missing bars (empty white space) represents stations/locations where samples were not collected at that particular depth. SRF, surface; MXL, mixed layer; DCM, deep chlorophyll maximum; MES, mesopelagic; IO, Indian Ocean; MS, Mediterranean Sea; NAO, North Atlantic Ocean; North Pacific Ocean; SAO, South Atlantic Ocean; SO, Southern Ocean; SPO, South Pacific Ocean.


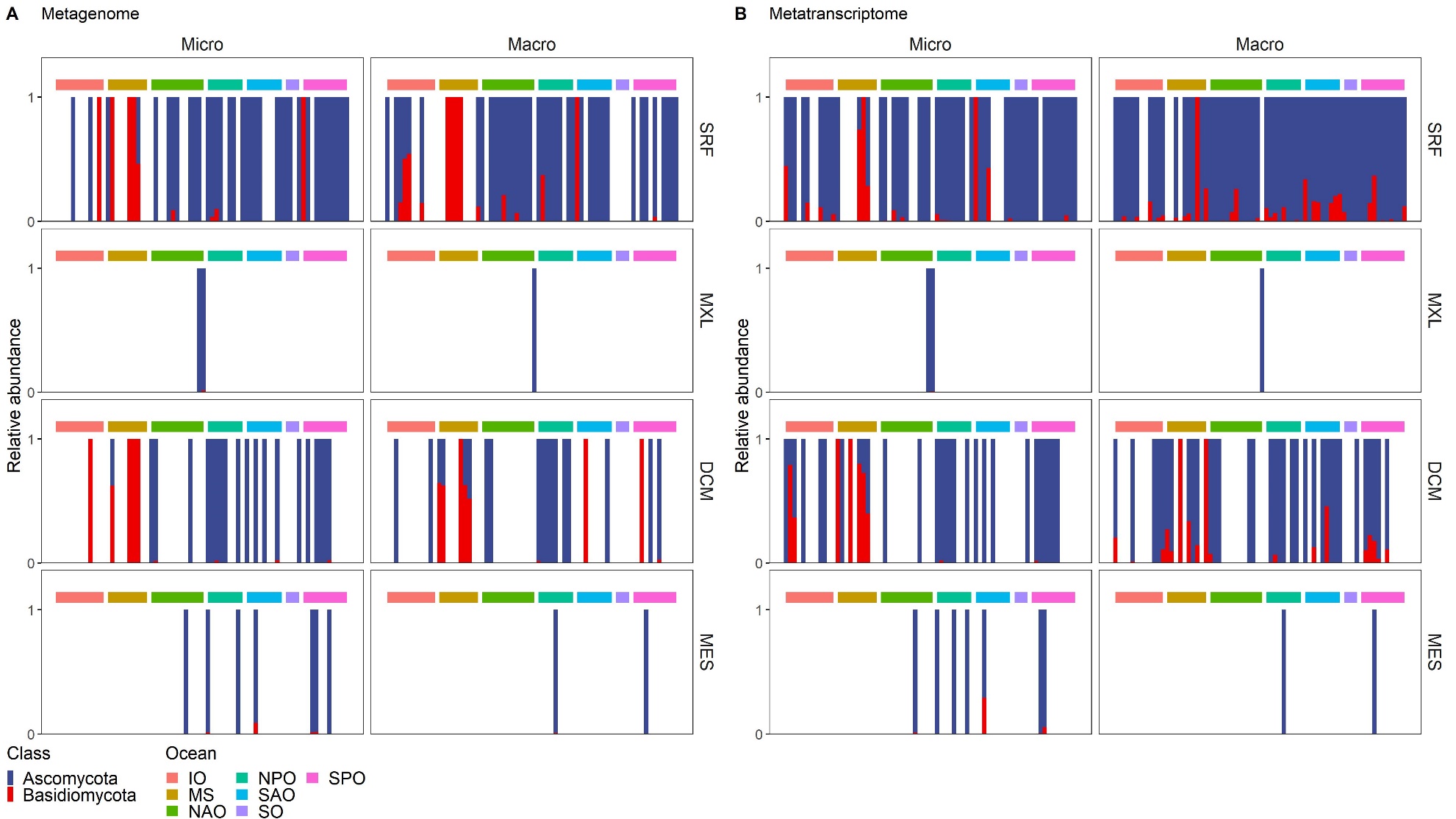


**Figure S4.** Taxonomic affiliation of genes (A) and transcripts (B) encoding secretory fungal CAZymes at the phylum level. Micro, micro-mycobiome (0.8-5 µm); Macro, macro-mycobiome (5-2,000 µm). Each bar represents a sample collected in each of the stations/location and depth; so that missing bars (empty white space) represents stations/locations where samples were not collected at that particular depth. SRF, surface; MXL, mixed layer; DCM, deep chlorophyll maximum; MES, mesopelagic; IO, Indian Ocean; MS, Mediterranean Sea; NAO, North Atlantic Ocean; North Pacific Ocean; SAO, South Atlantic Ocean; SO, Southern Ocean; SPO, South Pacific Ocean.


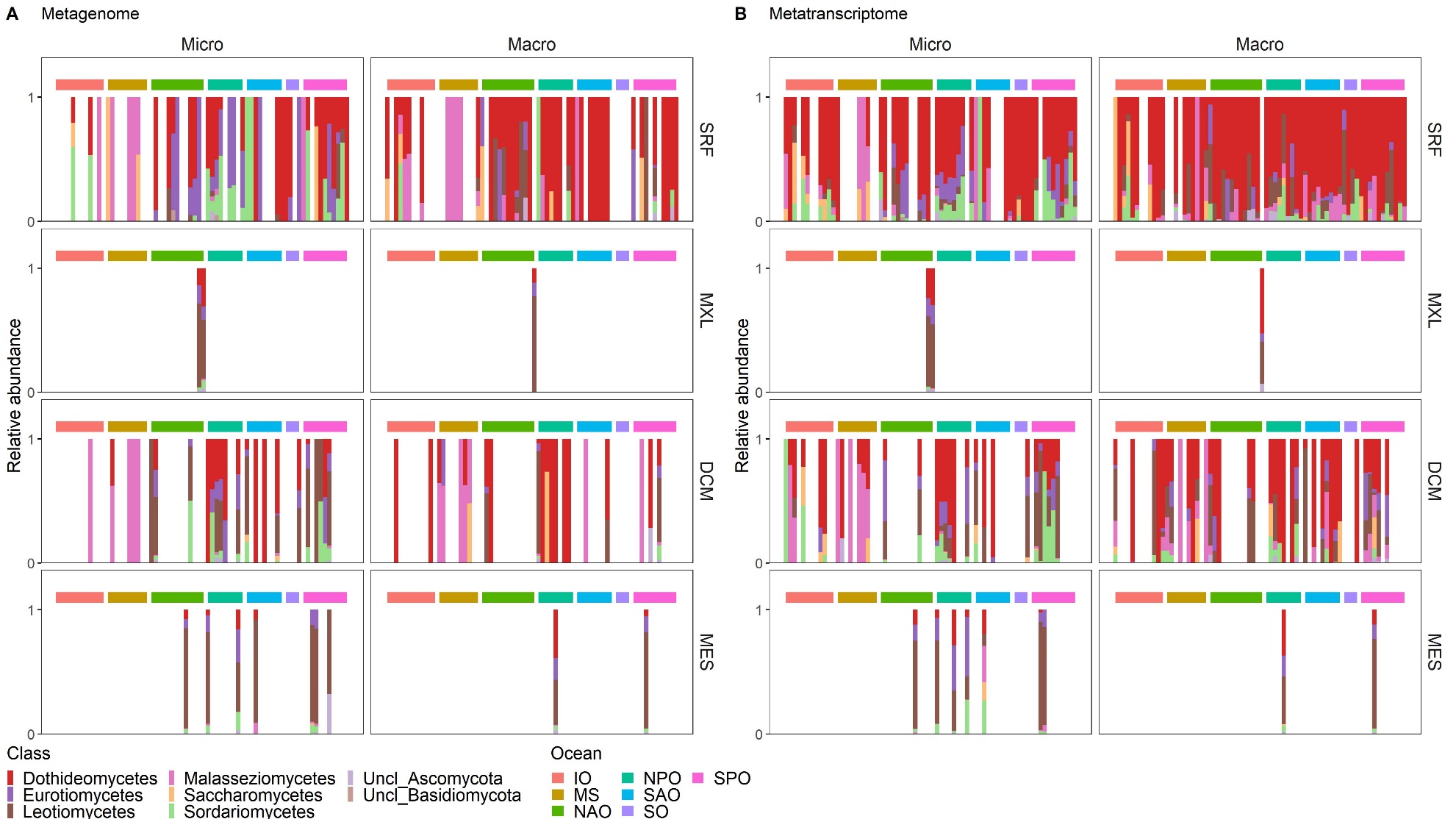


**Figure S5.** Taxonomic affiliation of genes (A) and transcripts (B) encoding secretory fungal CAZymes at the class level. Micro, micro-mycobiome (0.8-5 µm); Macro, macro-mycobiome (5-2,000 µm). Each bar represents a sample collected in each of the stations/location and depth; so that missing bars (empty white space) represents stations/locations where samples were not collected at that particular depth. SRF, surface; MXL, mixed layer; DCM, deep chlorophyll maximum; MES, mesopelagic; IO, Indian Ocean; MS, Mediterranean Sea; NAO, North Atlantic Ocean; North Pacific Ocean; SAO, South Atlantic Ocean; SO, Southern Ocean; SPO, South Pacific Ocean.


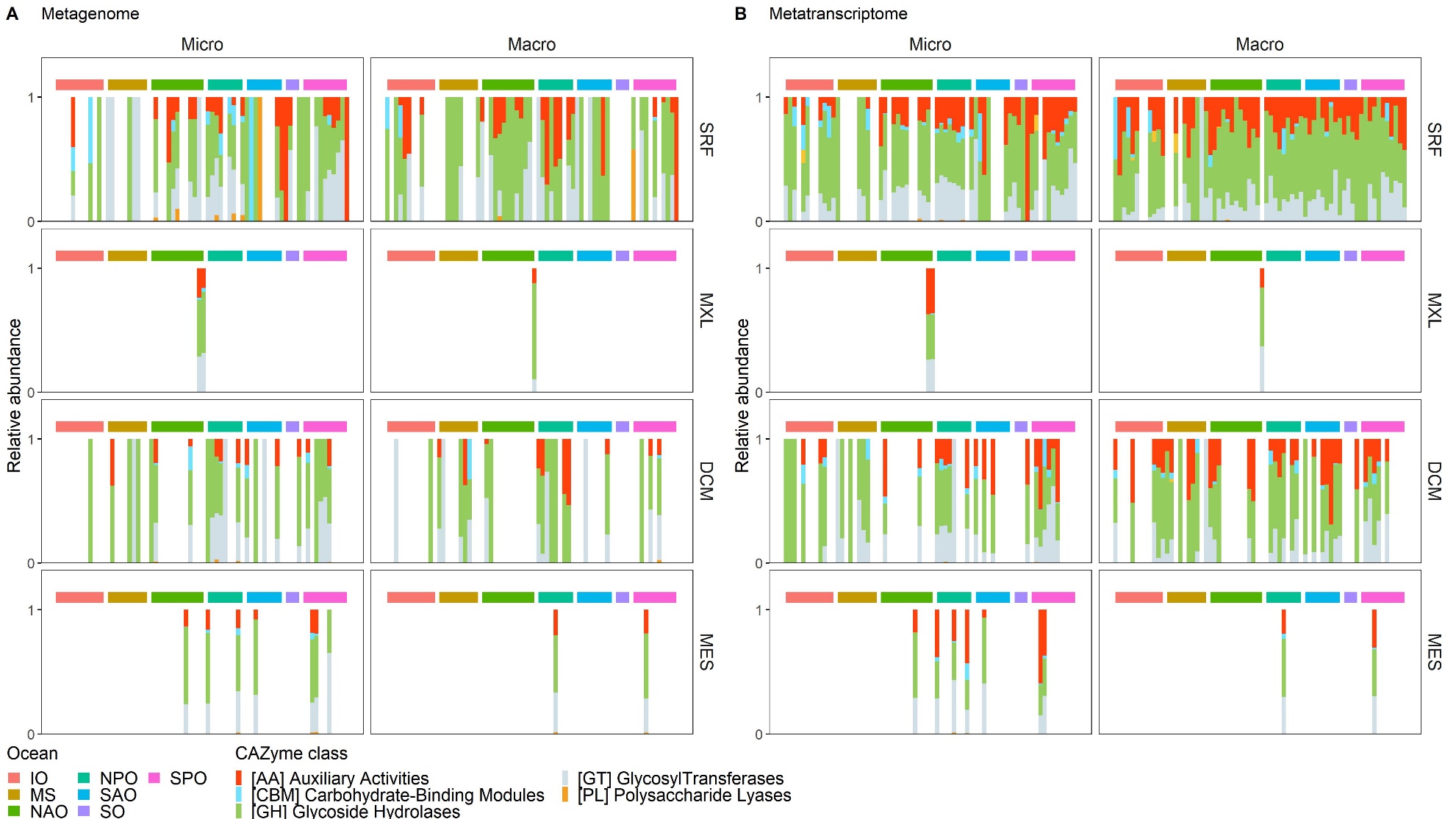


**Figure S6.** Functional classification of genes (A) and transcripts (B) encoding secretory fungal CAZymes. Micro, micro-mycobiome (0.8-5 µm); Macro, macro-mycobiome (5-2,000 µm). Each bar represents a sample collected in each of the stations/location and depth; so that missing bars (empty white space) represents stations/locations where samples were not collected at that particular depth. SRF, surface; MXL, mixed layer; DCM, deep chlorophyll maximum; MES, mesopelagic; IO, Indian Ocean; MS, Mediterranean Sea; NAO, North Atlantic Ocean; North Pacific Ocean; SAO, South Atlantic Ocean; SO, Southern Ocean; SPO, South Pacific Ocean.


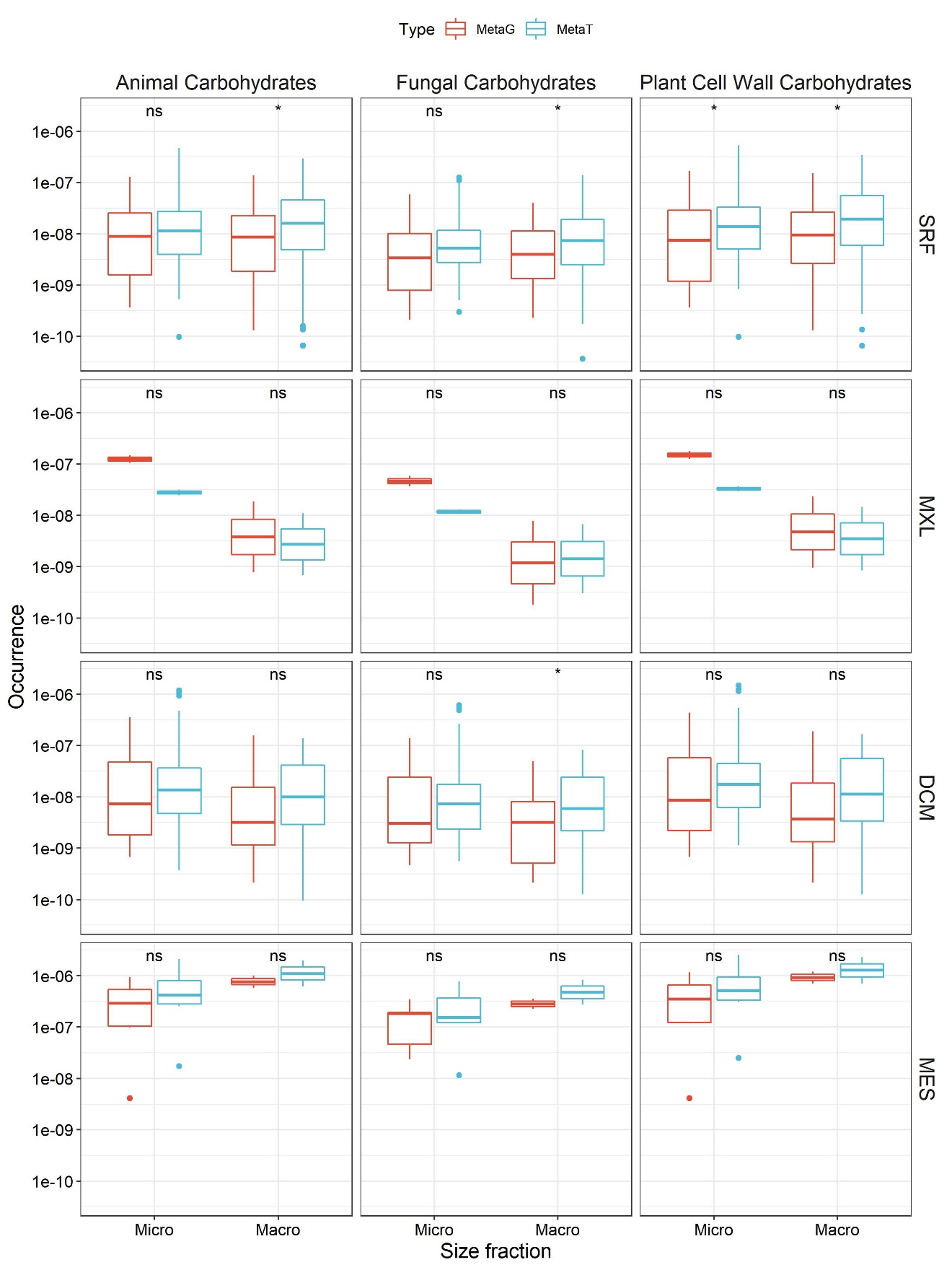


**Figure S7.** Occurrence of genes and transcripts for fungal CAZymes targeting different carbohydrate sources. Micro, micro-mycobiome (0.8-5 µm); Macro, macro-mycobiome (5-2,000 µm). Box shows median and interquartile range (IQR); whiskers show 1.5 × IQR of the lower and upper quartiles or range; outliers extend to the data range. Statistics are based on a Wilcoxon test, *P<0.05, **P<0.01, ***P<0.001, ****P<0.0001, ns, not significant. SRF, surface; MXL, mixed layer; DCM, deep chlorophyll maximum; MES, mesopelagic. Note that carbohydrates originating from bacterial (peptidoglycan) detritus were not plotted because they were not found in the metagenome or metatranscriptomes of pelagic fungi.


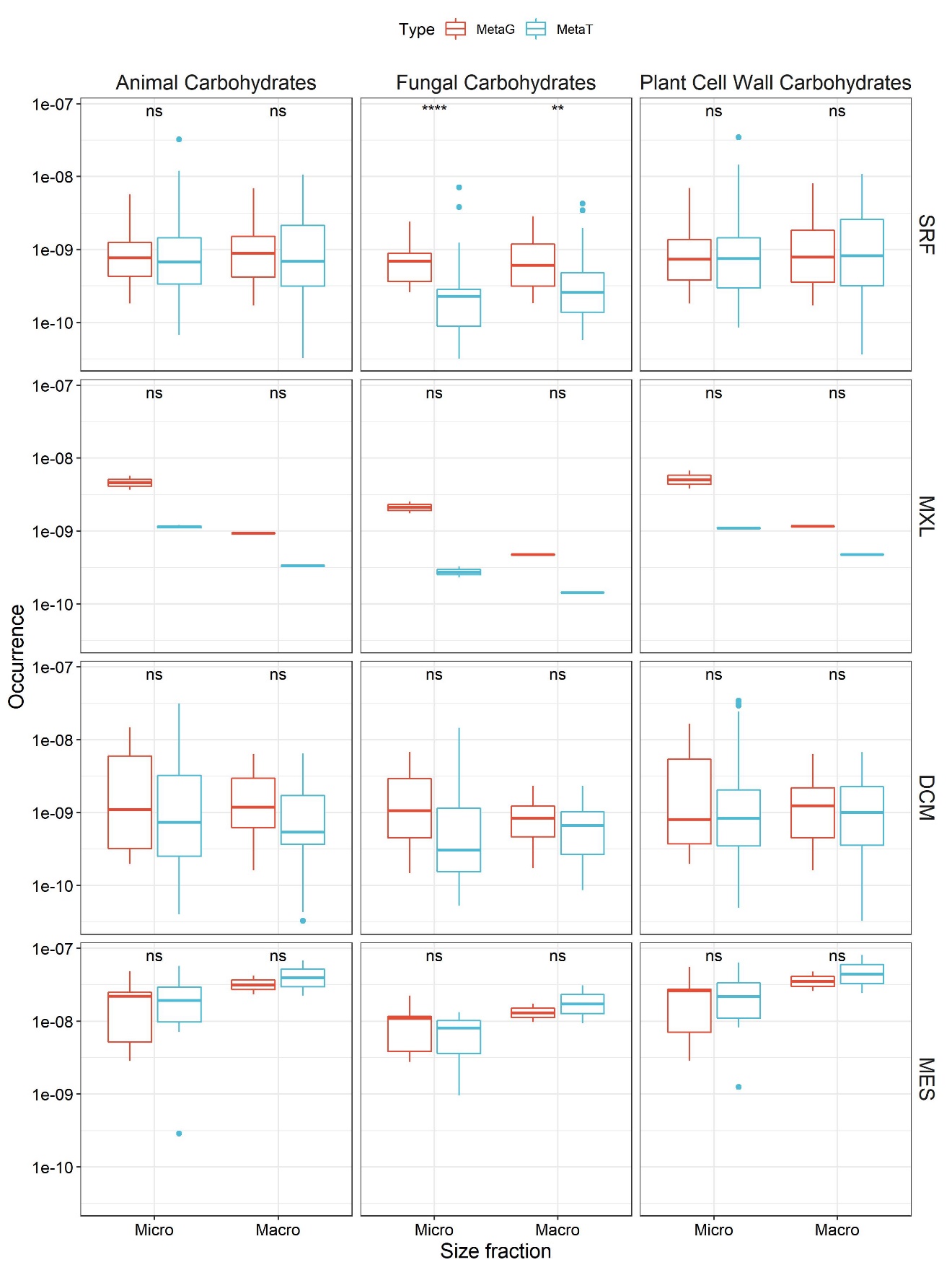


**Figure S8.** Occurrence of genes and transcripts for secretory fungal CAZymes targeting different carbohydrate sources. Micro, micro-mycobiome (0.8-5 µm); Macro, macro-mycobiome (5-2000 µm). Box shows median and interquartile range (IQR); whiskers show 1.5 × IQR of the lower and upper quartiles or range; outliers extend to the data range. Statistics are based on Wilcoxon test, *P<0.05, **P<0.01, ***P<0.001, ****P<0.0001, ns, not significant. SRF, surface; MXL, mixed layer; DCM, deep chlorophyll maximum; MES, mesopelagic.
